# African swine fever virus MGF360-11L negatively regulates cGAS-STING-mediated inhibition of type I interferon production

**DOI:** 10.1186/s13567-022-01025-0

**Published:** 2022-01-24

**Authors:** Kaidian Yang, Ying Xue, Hui Niu, Chunwei Shi, Mingyang Cheng, Jianzhong Wang, Boshi Zou, Junhong Wang, Tianming Niu, Meiying Bao, Wentao Yang, Dandan Zhao, Yanlong Jiang, Guilian Yang, Yan Zeng, Xin Cao, Chunfeng Wang

**Affiliations:** 1 College of Veterinary Medicine, Jilin Agricultural University, Changchun, China; 2 Jilin Provincial Key Laboratory of Animal Microecology and Healthy Breeding, Jilin Agricultural University, Changchun, China; 3 Jilin Provincial Engineering Research Center of Animal Probiotics, Jilin Agricultural University, Changchun, China; 4 Key Laboratory of Animal Production and Product Quality Safety of Ministry of Education, Jilin Agricultural University, Changchun, China

**Keywords:** MGF360-11L, IFN-I, signaling pathway, African swine fever virus

## Abstract

The type I interferon (IFN-I) signaling pathway is an important part of the innate immune response and plays a vital role in controlling and eliminating pathogens. African swine fever virus (ASFV) encodes various proteins to evade the host’s natural immunity. However, the molecular mechanism by which the ASFV-encoded proteins inhibit interferon production remains poorly understood. In the present study, ASFV MGF360-11L inhibited cGAS, STING, TBK1, IKKε, IRF7 and IRF3-5D mediated activation of the IFN-β and ISRE promoters, accompanied by decreases in IFN-β, ISG15 and ISG56 mRNA expression. ASFV MGF360-11L interacted with TBK1 and IRF7, degrading TBK1 and IRF7 through the cysteine, ubiquitin–proteasome and autophagy pathways. Moreover, ASFV MGF360-11L also inhibited the phosphorylation of TBK1 and IRF3 stimulated by cGAS-STING overexpression. Truncation mutation analysis revealed that aa 167-353 of ASFV MGF360-11L could inhibit cGAS-STING-mediated activation of the IFN-β and ISRE promoters. Finally, the results indicated that ASFV MGF360-11L plays a significant role in inhibiting IL-1β, IL-6 and IFN-β production in PAM cells (PAMs) infected with ASFV. In short, these results demonstrated that ASFV MGF360-11L was involved in regulating IFN-I expression by negatively regulating the cGAS signaling pathway. In summary, this study preliminarily clarified the molecular mechanism by which the ASFV MGF360-11L protein antagonizes IFN-I-mediated antiviral activity, which will help to provide new strategies for the treatment and prevention of ASF.

## Introduction

The pathogen that causes African swine fever (ASF) is an enveloped, double-stranded DNA virus that exists in the cytoplasm [1, 2]. ASF has caused serious economic losses to animal husbandry, the meat industry, and foreign trade exports [3–5]. ASF was classified as a category A disease of animals by the World Organization for Animal Health (OIE) and is listed as a category I disease of animals by China [6]. African swine fever virus (ASFV) can evade the host's defense system to combat the innate immune response through a complex interaction between the virus and the host [4, 7, 8]. There are currently no commercially available vaccines or drugs for the prevention and treatment of ASF [4]. Previous studies have reported that ASFV-encoded proteins could effectively inhibit cell and host defenses, which are essential for establishing immune evasion [9].

Evasion of host innate immunity plays a significant role in the pathogenesis of ASFV [7]. The innate immune response is the first line of defense against the invasion of microbial pathogens. After cells are infected with a DNA virus, a cytoplasmic sensor detects the viral DNA [10, 11]. Although a variety of cytoplasmic DNA sensors have been identified, cyclic GMP-AMP synthase (cGAS) has been widely accepted for detecting cytoplasmic DNA in a variety of cell types [12–14]. After viral DNA is detected, cGAS catalyzes the synthesis of the second messenger GMP-AMP (cGAMP), which is bound to stimulator of interferon gene (STING), a type I interferon (IFN-I) that is triggered by the virus and is a key adaptor for inducing of the innate antiviral response [15, 16]. STING is activated and translocates to the endoplasmic reticulum (ER), and then tank-binding kinase 1 (TBK1) kinase and the transcription factor interferon response factor 3 (IRF3) are recruited to promote the phosphorylation and dimerization of IRF3 in the ER. Finally, the expression of IFN-I and inflammatory factors is induced by IRF3 in the nucleus to activate the innate immune response [8, 17].

A recent study reported that China 2018/1 ASFV DP96R could inhibit the cGAS-STING-TBK1 signaling pathway [18]. ASFV multigene family (MGF) 360-12L blocked the interaction of importin a and the NF-κB signaling pathway to inhibit IFN-I production [12]. Moreover, ASFV MGF505-7R inhibited the cGAS-STING signaling pathway [19]. pMGF505-7R determines the pathogenicity of ASFV infection by inhibiting IL-1β and IFN-I production [20]. The molecular mechanism by which the immune escape protein of African swine fever virus evades the host’s defense system is still poorly understood [12]. Hence, in the present study, we explored the mechanism by which the ASFV immunosuppressive protein MGF360-11L regulates the cGAS-STING signaling pathway. We discovered that ASFV MGF360-11L plays a significant role in inhibiting the IFN-I signaling pathway. To the best of our knowledge, this is the first report to show that ASFV MGF360-11L inhibits IFN-I production by regulating the cGAS-STING signaling pathway. ASFV MGF360-11L interacted with TBK1 and IRF7 and inhibited IFN-I production. These findings could provide a reference for further elucidation of viral immune evasion mechanisms and the development of safe and effective vaccines to prevent ASF.

## Materials and methods

### Cells and viruses

Porcine kidney (PK)-15, 3D4/21 and human embryonic kidney (HEK)-293 T cells were purchased from the American Type Culture Collection (ATCC) and cultured in RPMI-1640 (Gibco, USA) medium containing 10% fetal bovine serum (FBS; Gibco) in 5% CO_2_ at 37 °C. To prepare PAM cells (PAMs) as described by Li et al. [21], PAMs were isolated from the lung lavage fluid of specific pathogen-free (SPF) piglets aged 4 weeks. The cells were cultured in complete RPMI-1640 at 37 °C with 5% CO_2_. Herpes simplex virus (HSV) was stored in our laboratory. ASFV (SY18 strain, GenBank: MH766894) was isolated from pigs as previously described [22].

### Fifty percent hemadsorption dose (HAD50) assay

ASFV was quantified by HAD50 assays according to previously described methods [22]. Briefly, PAMs were seeded in a 96-well plate and infected with tenfold serial dilutions of virus. At 7 days after infection, the HAD50 was determined using the Reed-Muench method. All data are shown as the means of three independent experiments.

### Antibodies and reagents

#### Antibodies

Anti-GAPDH antibodies, anti-Flag agarose affinity gels, and anti-HA agarose affinity gels were supplied by Sigma–Aldrich (Shanghai, China). Mouse anti-Flag-horseradish peroxidase (HRP), anti-HA-HRP and anti-Myc-HRP antibodies were purchased from Roche (Basel, Switzerland). HRP-conjugated goat anti-mouse IgG (H + L) was obtained from Proteintech Group, Inc. (Rosemont, IL, USA).

#### Reagents

The double-luciferase reporter assay kit was provided by Promega (Madison, USA). The plasmid prep purification kit was purchased from Omega (Georgia, USA). The MiniBEST universal RNA extraction kit, SYBR green qPCR mix and PrimeScript™ RT reagent kit with gDNA eraser were purchased from TaKaRa (Dalian, China). Lipofectamine 3000 was supplied by Thermo Fisher (Grand Island, USA). Radioimmunoprecipitation assay (RIPA) buffer, DMSO, MG132, and 3-methyladenine (3-MA) were provided by Sigma–Aldrich (Shanghai, China). Polyvinylidene fluoride (PVDF) membranes were purchased from Merck Millipore (Burlington, USA). Bovine serum albumin (BSA) and 5 × SDS loading sample buffer were purchased from Solarbio (Beijing, China). Swine IFN-β/IL-1β/IL-6 commercial ELISA kits were supplied by Thermo Fisher (Grand Island, USA).

### Construction and transfection of plasmids

The MGF360-11L gene of ASFV SY18 (GenBank: MH766894) was synthesized and cloned into the pCMV-*N*-HA vector with EcoR I and Not I sites by using standard molecular biology techniques [23]. The ASFV MGF360-11L truncation mutants, including MGF360-11L-1 (1-180 aa) and MGF360-11L-2 (167-353 aa), were cloned into the pCMV-*N*-HA vector. Plasmids for Flag-tagged cGAS, STING, TRAF3, TRAF6, TBK1, IKKi, IKKε, IRF3, IRF7, and IRF3-5D, and the IFN-β-Luc and ISRE-Luc luciferase reporter plasmids, as well as pRL-TK, were described previously [18, 19]. The plasmid DNA of recombinant bacteria was extracted using a plasmid prep purification kit (Omega). The procedure was performed according to the manufacturer's instructions. Briefly, recombinant bacteria were incubated for 16 h at 37 °C with shaking and centrifuged at 10 000 × *g* for 1 min at room temperature (RT) to collect bacterial pellets. Solution I/RNase, solution II, N3 buffer, ETR binding buffer, ETR wash buffer, HBC buffer, and DNA wash buffer were added, the sample was centrifuged, and the filtered liquid was discarded. Finally, the elution buffer was added and centrifuged to collect the filtered liquid. Plasmids in the filtered liquid were stored at −20 °C. Purified plasmids were co-transfected with Lipofectamine™ 3000 transfection reagent at a ratio of 2:1 to 3:1 Lipofectamine™ 3000/DNA into HEK-293 T cells and PK-15 cells, respectively, and then the cells were cultured in fresh complete DMEM at 37 °C for 24 h for subsequent experiments.

### RT‑PCR

Total RNA was extracted from the indicated cells, and 1 μg of RNA was reverse transcribed to cDNA with a PrimeScript™ RT reagent kit. RT–PCR was conducted with 1 μL of cDNA as a template using SYBR green qPCR mix (Takara) in an AB 7500 system (Applied Biosystems, USA). GAPDH was used as the endogenous control. Human and pig GAPDH were used for normalization, and the results were calculated using 2^−∆∆CT^ method [24]. All samples were analyzed in triplicate. The primers used in this study are described in Table 1.

**Table 1:** **The primer sequences for RT-PCR**.

Primers	Sequence (5′ → 3′)
Human IFN-β-forward	GCTTGGATTCCTACAAAGAAGCA
Human IFN-β-reverse	ATAGATGGTCAATGCGGCGTC
Human ISG15-forward	CGCAGATCACCCAGAAGATCG
Human ISG15-reverse	TTCGTCGCATTTGTCCACCA
Human ISG56-forward	TTGATGACGATGAAATGCCTGA
Human ISG56-reverse	CAGGTCACCAGACTCCTCAC
Human GAPDH-forward	GGAGCGAGATCCCTCCAAAAT
Human GAPDH-reverse	GGCTGTTGTCATACTTCTCATGG
Pig IFN-β-forward	GCTAACAAGTGCATCCTCCAAA
Pig IFN-β-reverse	AGCACATCATAGCTCATGGAAAGA
Pig ISG15-forward	GATCGGTGTGCCTGCCTTC
Pig ISG15-reverse	CGTTGCTGCGACCCTTGT
Pig ISG56-forward	AAATGAATGAAGCCCTGGAGTATT
Pig ISG56-reverse	AGGGATCAAGTCCCACAGATTTT
Pig P72-forward	CCCAGGRGATAAAATGACTG
Pig P72-reverse	CACTRGTTCCCTCCACCGATA
Pig GAPDH-forward	ACATGGCCTCCAAGGAGTAAGA
Pig GAPDH-reverse	GATCGAGTTGGGGCTGTGACT
Pig MGF360-11L-forward	GCGGTGGACTATGACCTCAAAGATG
Pig MGF360-11L-reverse	TGCGGACCCTTTCTATTTCGTACAG

### Dual-luciferase reporter assays

The reporter plasmids IFN-β-Luc (100 ng) and ISRE-Luc (100 ng) were respectively co-transfected with the pRL-TK plasmid (10 ng) and the indicated plasmids or empty vector plasmid (pCMV-*N*-HA) into HEK-293 T cells using Lipofectamine™ 3000 (Thermo Fisher). Twenty-four hours after transfection, the cells were treated with 1 × passive lysis buffer (PLB) (Promega) for 20 min at RT with shaking and then centrifuged to collect the supernatants, and the activities of firefly and Renilla luciferase were determined using a dual-luciferase reporter assay system (Promega). All experiments were independently repeated at least three times.

### Western blotting

The indicated plasmids were co-transfected into HEK-293 T cells. At 24 h post-transfection, the cells were lysed with RIPA buffer. Total cell protein was collected and boiled for 10 min, 5 × SDS loading buffer (Solarbio) was added, and the samples were centrifuged (10 000 × *g*, 10 min, 4 °C). Then, each sample was subjected to SDS–PAGE followed by transfer onto PVDF membranes (Merck Millipore). The membranes were blocked with 5% BSA (Solarbio) for 2 h at RT and then incubated with a specific primary antibody at 4 °C for 12 h with shaking. After being washed with PBST (0.5% Tween 20), the membranes were incubated with secondary antibodies for 2 h at RT. After being washed using PBST, protein bands were observed and imaged by an Amersham Imager 600 RGB (GE, Marlborough, USA). The following antibodies were used: mouse anti-HA-HRP (1:1000 dilution), mouse anti-Flag-HRP (1:1000 dilution), mouse anti-Myc-HRP (1:2000 dilution), mouse anti-GAPDH (1:3000 dilution), rabbit anti-IRF3 (1:1000 dilution), rabbit anti-TBK1 (1:1000 dilution), phospho-TBK1 (Ser172) (1:1000 dilution), and phospho-IRF3 (Ser386) (1:1000 dilution).

### Co-immunoprecipitation (Co-IP)

The indicated plasmids were transfected into HEK-293 T cells. After 24 h of transfection, the cells were harvested and lysed with IP lysis buffer. For each IP reaction, 1 mL of lysate was co-incubated with anti-Flag/HA agarose affinity gel (Sigma–Aldrich) for 12 h at 4 °C with shaking. After being washed with PBST, the beads were incubated with 1 mL of lysis buffer and used for the Co-IP assay. Immunoprecipitation was followed by Western blotting with anti-Flag and anti-HA antibodies.

### siMGF360-11L-mediated knockdown

The primers for MGF360-11L siRNAs are listed in Table 2. siRNA transfection was conducted using jetPEI^®^-macrophage in vitro DNA transfection reagent (Polyplus) according to the instructions. SiMGF360-11L was transfected into PAMs for 24 h, and the cells were infected with ASFV (MOI = 1) for another 12 h or 24 h. IFN-β, ISG15 and ISG56 mRNA levels were measured by RT–PCR. The cytokines IFN-β, IL-1β and IL-6 in the cell culture supernatants were measured by commercial ELISA kits (Thermo Fisher). RT-PCR and Western blotting was used to measure the expression level of the P72 protein.

**Table 2:** **siRNA sequences used in this study**.

Primers	Sequence (5′ → 3′)
siMGF360-11L-forward	CAAAUACUGGUACGCGAUAdTdT
siMGF360-11L-reverse	UAUCGCGUACCAGUAUUUGdTdT
siNC-forward	UUCUCCGAACGUGUCACGUTT
siNC-reverse	ACGUGACACGUUCGGAGAATT

### Statistical analysis

The data in the present study were processed with GraphPad Prism 8 software, and the results are expressed as the arithmetic means ± standard deviation. Intragroup or intergroup differences were analyzed by Student’s *t* test or one-way ANOVA, with at least three independent trials. *P < 0.05, **P < 0.01, and ***P < 0.001 were defined as statistically significant.

## Results

### ASFV MGF360-11L inhibits activation of the IFN-β and ISRE promoters

**Figure 1:**
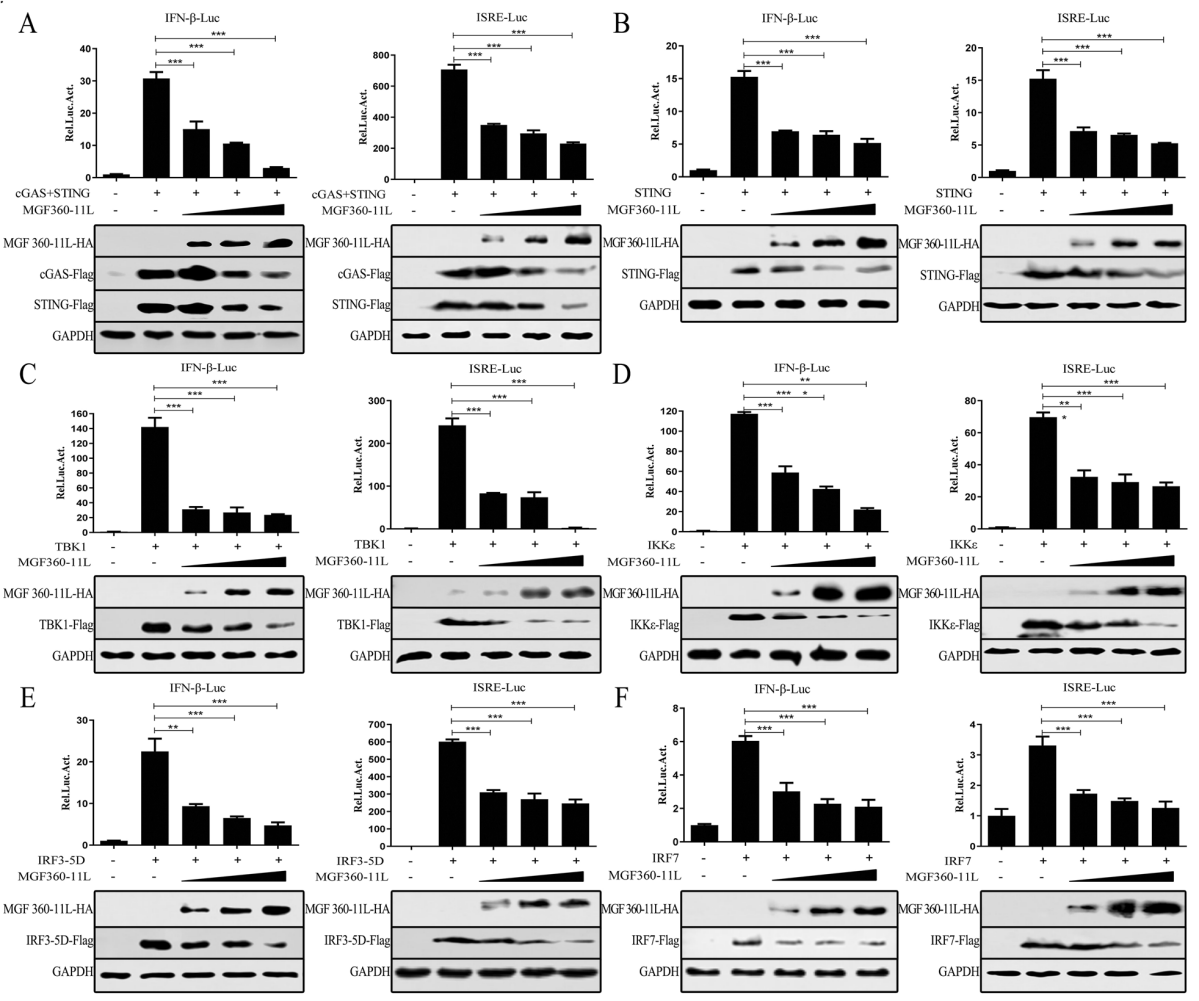
**ASFV MGF360-11L inhibited the activation of IFN-β and the ISRE promoter.** HEK-293 T cells were co-transfected with IFN-β-Luc (100 ng), ISRE-Luc (100 ng), and pRL-TK (10 ng) plasmids and the IFN-I signaling molecule plasmids cGAS (100 ng, **A**), STING (100 ng, **A**), STING (200 ng, **B**), TBK1 (200 ng, **C**), IKKε (200 ng, **D**), IRF3-5D (200 ng, **E**), and IRF7 (200 ng, **F**), along with increasing doses of MGF360-11L (50, 100, 200 ng) plasmid or empty vector plasmid (pCMV-*N*-HA). 24 h post-transfection, cell lysates were used for dual-luciferase reporter assays. The expression of cGAS, STING, TBK1, IKKε, IRF, IRF7 and MGF360-11L was analyzed by Western blotting. All assays were independently repeated at least three times. The data are shown as the mean ± SD; *n* = 3. *p < 0.05, **p < 0.01, ***p < 0.001. Luc: luciferase.

To explore whether ASFV MGF360-11L could regulate factors in the cGAS-STING signaling pathway, we evaluated the effect of ASFV MGF360-11L on cGAS, STING, TBK1, IRF3-5D (an active form of IRF3), IKKε (a constitutively active form of IKKi) and IRF7 expression by dual-luciferase reporter assays. The results revealed that ASFV MGF360-11L inhibited IFN-β and ISRE activation because MGF360-11L inhibited the expression of cGAS/STING (Figure 1A), STING (Figure 1B), TBK1 (Figure 1C), IRF3-5D (Figure 1D), IKKε (Figure 1E) and IRF7 (Figure 1F) in a dose-dependent manner in HEK-293 T cells, indicating that ASFV MGF360-11L efficiently inhibited the IFN-I response by regulating the cGAS-STING pathway.

### Effect of ASFV MGF360-11L on the transcription of IFN-β and ISGs

**Figure 2:**
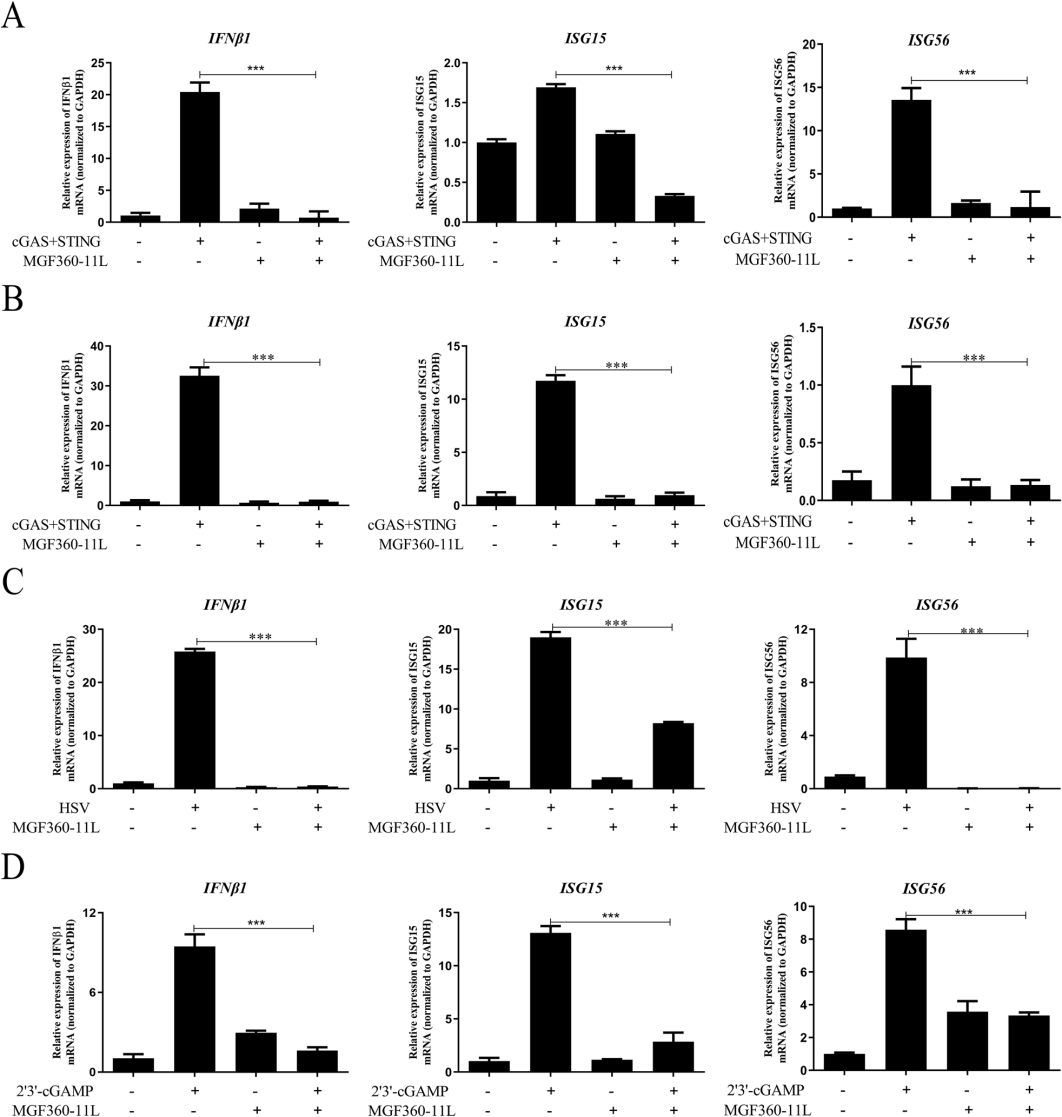
**ASFV MGF360-11L could inhibit the IFN-β signaling pathway.** cGAS (500 ng) and STING (500 ng) plasmids were co-transfected into HEK-293 T cells (**A**) and PK-15 cells (**B**), along with MGF360-11L (1 μg) or empty vector plasmids. Twenty-four hours post-transfection, IFN-β, ISG15, and ISG56 mRNA was analyzed by RT–PCR. **C** MGF360-11L (1 μg) or empty vector plasmids were co-transfected into PK-15 cells for 18 h, and then the cells were infected with HSV (MOI = 1) for another 8 h. RT–PCR was conducted with the indicated primers. **D** MGF360-11L (1 μg) or empty vector plasmids were co-transfected into 3D4/21 cells, after 24 h post-transfection cells were stimulated with 2′3′-cGAMP for 12 h, RT–PCR was conducted with the indicated primers. All experiments were independently repeated at least 3 times. The data are shown as the mean ± SD; *n* = 3. *p < 0.05, **p < 0.01, ***p < 0.001.

We examined the effect of ASFV MGF360-11L on the inhibition of the IFN-I downstream antiviral response. The results showed that ASFV MGF360-11L could inhibit cGAS-STING-induced transcription of the IFN-β, ISG15, and ISG56 genes in HEK-293 T cells (Figure 2A). Similarly, ASFV MGF360-11L also inhibited the transcription of the IFN-β, ISG15, and ISG56 genes triggered by cGAS-STING in PK-15 cells (Figure 2B). Further analysis indicated that in PK-15 cells infected with HSV, ASFV MGF360-11L inhibited transcription of the IFN-β, ISG15 and ISG56 genes (Figure 2C). ASFV MGF360-11L inhibited transcription of the IFN-β, ISG15 and ISG56 genes in 3D4/21 cells when stimulated with 2′3′-cGAMP (Figure 2D). These results revealed that ASFV MGF360-11L could inhibit the antiviral response of IFN-I downstream genes.

### ASFV MGF360-11L interacts with TBK1, IRF7

**Figure 3:**
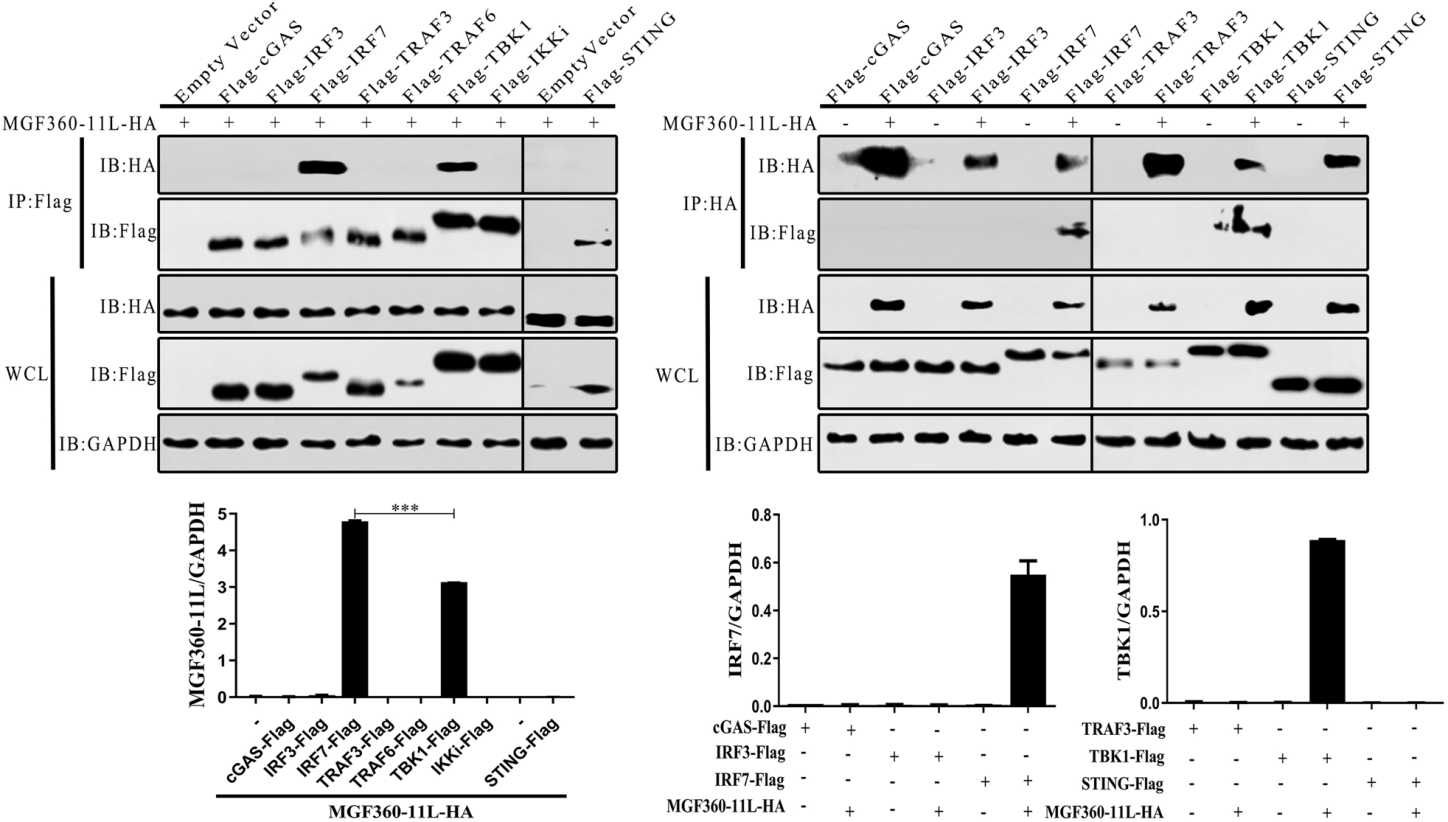
**ASFV MGF360-11L could interact with TBK1 and IRF7.** The MGF360-11L-HA (1 μg) plasmid was co-transfected with Flag-tagged IFN-I signaling molecule plasmids for cGAS (1 μg), STING (1 μg), TRAF3 (100 ng), TRAF6 (200 ng), TBK1 (1 μg), IKKi (500 ng), IRF3 (500 ng) and IRF7 (1 μg) into HEK-293 T cells, followed by IP with anti-Flag agarose affinity gel. The MGF360-11L-HA plasmid (1 μg) was co-transfected with Flag-tagged IFN-I signaling molecule plasmids for cGAS (1 μg), STING (1 μg), TRAF3 (100 ng), TBK1 (500 ng), IRF3 (200 ng) and IRF7 (1 μg) into HEK-293 T cells, followed by IP with anti-HA agarose affinity gel. Co-IP and Western blotting were carried out using anti-HA-HRP and anti-flag-HRP antibodies. All experiments were independently repeated at least three times. The data are shown as the mean ± SD; *n* = 3. *p < 0.05, **p < 0.01, ***p < 0.001. IB, immunoblotting; HA, anti-HA-tagged monoclonal antibody; IP, immunoprecipitation; WCL, whole cell lysate.

To explore the mechanisms of ASFV MGF360-11L in the innate immune response, changes in cGAS, STING, TRAF3, TRAF6, TBK1, IKKi, IRF3 and IRF7 were investigated in response to MGF360-11L. However, it is unclear which molecule in the IFN-I pathway might interact with ASFV MGF360-11L to exert its biological effects. Hence, cGAS, STING, TRAF3, TRAF6, TBK1, IKKi, IRF3 and IRF7 plasmids were co-transfected with MGF360-11L plasmids into HEK-293T cells, respectively (Figure 3). The Co-IP results confirmed that TBK1 and IRF7 could interact with MGF360-11L to inhibit IFN-I expression.

### ASFV MGF360-11L inhibits the phosphorylation of TBK1 and IRF3

**Figure 4:**
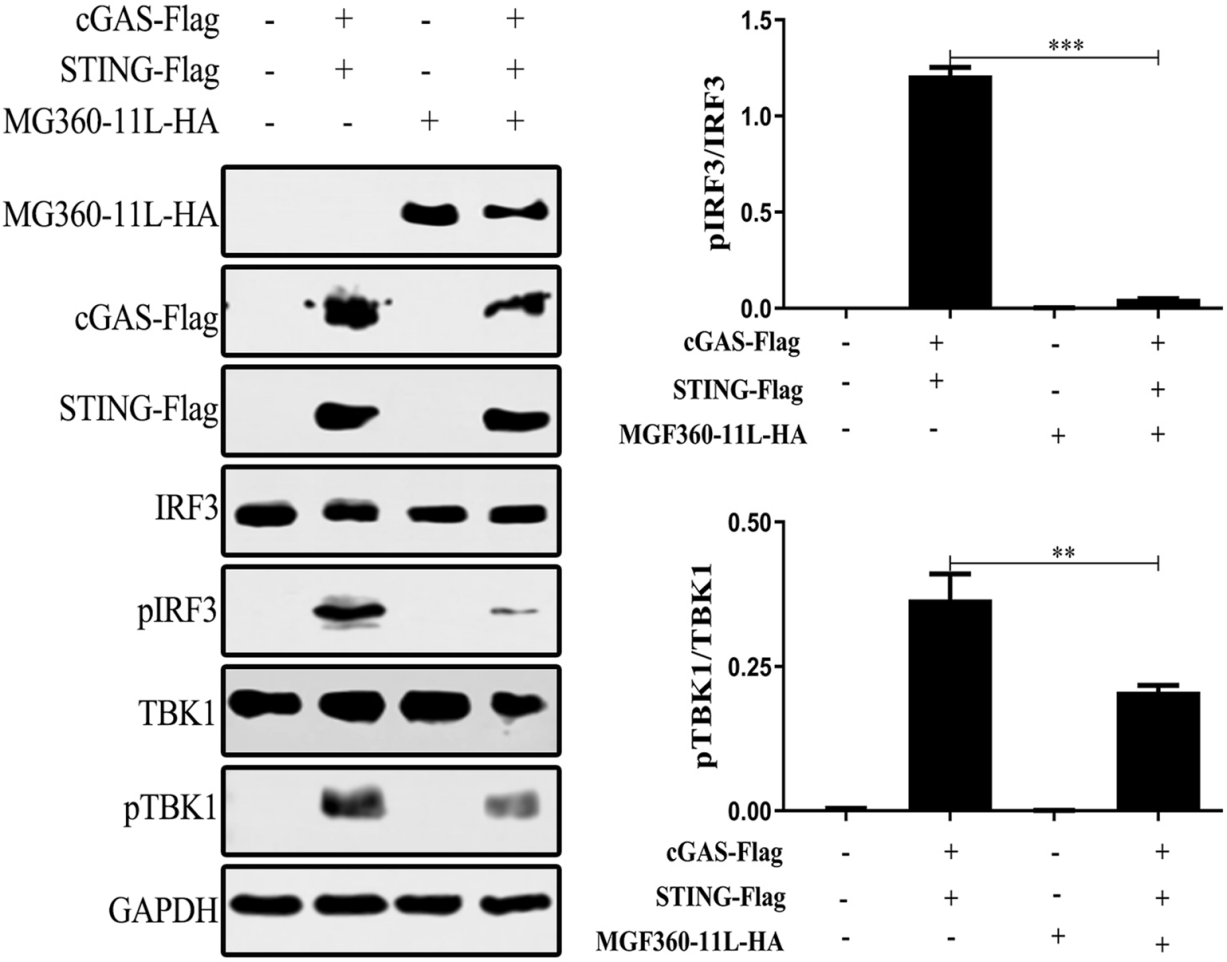
**ASFV MGF360-11L inhibited cGAS-STING-induced TBK1 and IRF3 phosphorylation.** The MGF360-11L (1 μg) plasmid was co-transfected with cGAS (500 ng) and STING (500 ng) plasmids into HEK-293 T cells. At 24 h post-transfection, the cells were lysed with RIPA buffer, and the phosphorylation of IRF3 and TBK1 was measured by Western blotting. All experiments were independently repeated at least 3 times. The data are shown as the mean ± SD; *n* = 3. *p < 0.05, **p < 0.01, ***p < 0.001.

TBK1 and IRF3 are vital transcription factors for IFN-I production. The phosphorylation of IRF3 and TBK1 is a hallmark of IRF3 and TBK1 activation. To determine whether ASFV MGF360-11L suppressed IFN-I production by phosphorylating TBK1 or IRF3, plasmids encoding cGAS and STING were co-transfected with the MGF360-11L plasmid into HEK-293T cells, and the results revealed that ASFV MGF360-11L could block the phosphorylation of TBK1 and IRF3 stimulated by cGAS-STING overexpression (Figure 4). These results suggested that ASFV MGF360-11L suppressed the IFN-I immune response by reducing the phosphorylation of TBK1 and inhibiting the downstream activation of IRF3.

### ASFV MGF360-11L degrades TBK1 and IRF7

**Figure 5:**
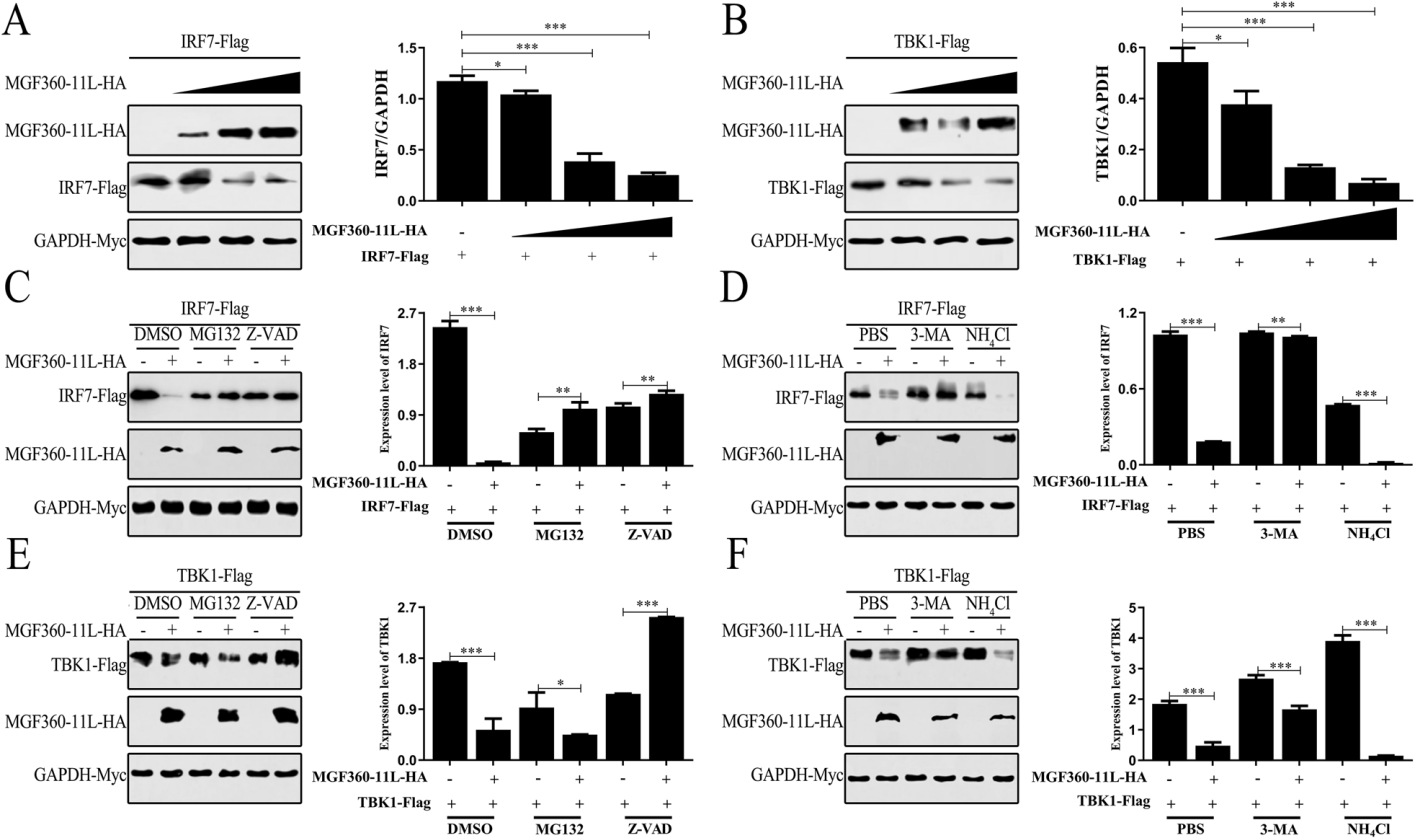
**Influence of inhibitors on ASFV MGF360-11L the degradation of TBK1 and IRF7.** HEK-293 T cells were co-transfected with IRF7 (200 ng, **A**) or TBK1 (200 ng, **B**) plasmids, Myc-GAPDH (100 ng), along with increasing doses of MGF360-11L (0, 100, 200, 400 ng) plasmids. 24 h post-transfection, Western blot analysis was performed with the indicated antibody. MGF360-11L (500 ng) and Myc-GAPDH (100 ng) plasmids were transfected with TBK1 (500 ng) and IRF7 (500 ng) plasmids into HEK-293 T cells, respectively. After 18 h of transfection, the cells were incubated with 3-MA (10 mM), NH_4_Cl (20 mM), MG132 (10 μM) and Z-VAD (20 μM) for 6 h. The cells were analyzed by Western blotting using the indicated antibody (**C**–**F**). All experiments were independently repeated at least three times. The data are shown as the mean ± SD; *n* = 3. *p < 0.05, **p < 0.01, ***p < 0.001.

Experimental evidence has demonstrated that ASFV MGF360-11L can interact with TBK1 and IRF7 (Figures 3A and B). We were interested in whether ASFV MGF360-11L could affect the expression of TBK1 and IRF7. TBK1 and IRF7 plasmids were transfected with the ASFV MGF360-11L plasmid into HEK-293 T cells. Western blot analysis revealed that the overexpression of ASFV MGF360-11L led to decreased TBK1 and IRF7 expression (Figures 5A and B), indicating that ASFV MGF360-11L could degrade TBK1 and IRF7.

To further explore the mechanism by which ASFV MGF360-11L affected the stability of TBK1 and IRF7, HEK-293 T cells were co-transfected with the indicated plasmids for 24 h and treated with various inhibitors of the protein degradation pathway (Figures 5C–F). We found that ASFV MGF360-11L-induced TBK1 and IRF7 degradation was inhibited by the autophagosome inhibitor 3-MA, cysteine inhibitor Z-VAD and proteasome inhibitor MG132. These results suggested that ASFV MGF360-11L could inhibit the IFN-I signaling pathway by degrading TBK1 and IRF7.

### The domains responsible for ASFV MGF360-11L inhibitory activity

**Figure 6:**
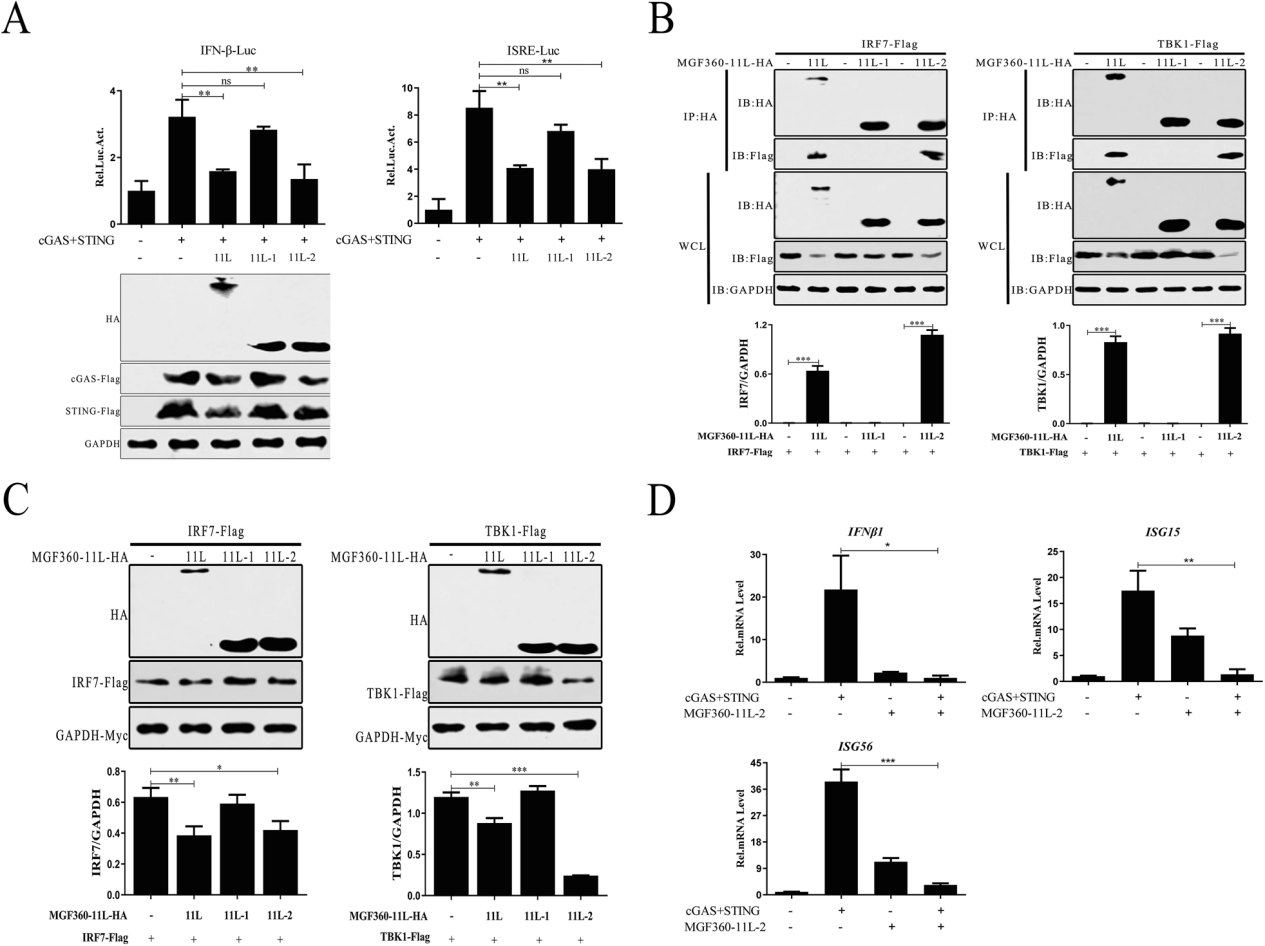
**The N-terminal domain of ASFV MGF360-11L exerted its inhibitory function.** **A** HEK-293 T cells were co-transfected with IFN-β-Luc (100 ng), ISRE-Luc (100 ng), pRL-TK (10 ng), cGAS (100 ng), and STING (100 ng) plasmids, full-length MGF360-11L and its truncated plasmids (MGF360-11L-1, MGF360-11L-2) for 24 h, and cell lysates were used for dual-luciferase reporter assays. **B**, **C** HEK-293T cells were co-transfected with TBK1 (1 μg) or IRF7 (1 μg) plasmid, along with full-length MGF360-11L (1 μg) plasmid and its truncated plasmids (MGF360-11L-1, MGF360-11L-2) for 24 h. Cells were analyzed by Co-IP and Western blotting with the indicated antibodies. **D** cGAS (500 ng) and STING (500 ng) plasmids were co-transfected into HEK-293T cells, along with the MGF360-11L-2 (1 μg) plasmid. Twenty-four hours post-transfection, RT–PCR was performed to determine IFN-β, ISG15, and ISG56 mRNA levels. All experiments were independently repeated at least 3 times. The data are shown as the mean ± SD; *n* = 3. *p < 0.05, **p < 0.01, ***p < 0.001. IB, immunoblotting; HA, anti-HA-tagged monoclonal antibody; IP, immunoprecipitation; WCL, whole cell lysate. 11L: MGF360-11L; 11L-1: MGF360-11L-1 (1-180 aa); 11L-2: MGF360-11L-2 (167-353 aa).

To further determine the domains responsible for ASFV MGF360-11L-mediated inhibitory activity, the ASFV MGF360-11L functional domain was truncated into two segments: MGF360-11L-1 (1-180 aa) and MGF360-11L-2 (167-353 aa). cGAS and STING plasmids were co-transfected with the truncated mutants of ASFV MGF360-11L into HEK-293T cells and examined by dual-luciferase reporter assays and Western blotting. The results indicated that ASFV MGF360-11L and MGF360-11L-2 could inhibit activation of the IFN-β promoter by cGAS-STING in HEK-293T cells (Figure 6A). In addition, we also found that the interaction of ASFV MGF360-11L and MGF 360-11L-2 with TBK1 and IRF7 decreased the expression of TBK1 and IRF7 (Figures 6B and C). ASFV MGF360-11L-2 inhibited cGAS-STING-induced transcription of the IFN-β, ISG15 and ISG56 genes in HEK-293 T cells (Figure 6D). ASFV MGF360-11L and MGF360-11L-2 inhibited the IFN-I response, which was related to TBK1 and IRF7.

### ASFV MGF360-11L inhibits the expression of IL-1β, IL-6 and IFN-β in PAMs infected with ASFV

**Figure 7:**
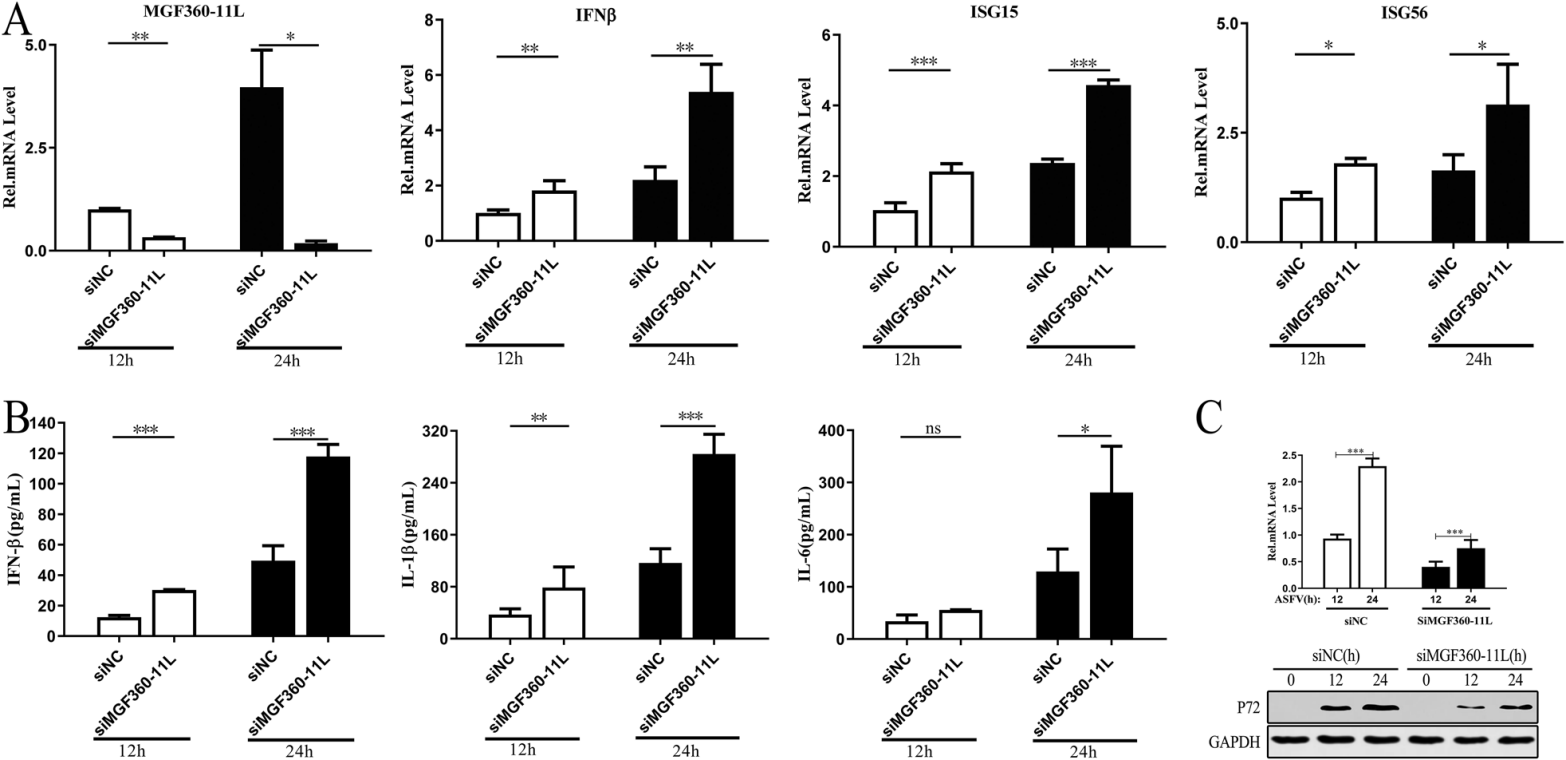
**The effect of siMGF360-11L on the expression of IFN-β, IL-1β and IL-6.** PAMs were transfected with siMGF360-11L or nontargeting control siRNA and infected with ASFV at an MOI of 1.0 for 12 h or 24 h. **A** RT–PCR was conducted to measure IFN-β, ISG15, and ISG56 mRNA levels. **B** ELISA was carried out to measure the secretion of the cytokines IFN-β, IL-1β and IL-6 in the cell supernatant. **C** RT–PCR and Western blotting were performed to measure the expression level of P72. All experiments were independently repeated at least three times. The data are shown as the mean ± SD; *n* = 3. *p < 0.05, **p < 0.01, ***p < 0.001.

To verify the effect of ASFV MGF360-11L on inhibiting the IFN-I antiviral response, specific siRNAs against MGF360-11L and nontargeting control siRNA were synthesized and transfected into PAMs, and the cells were infected with ASFV at an MOI of 1.0 for 12 h or 24 h. The results showed that ASFV MGF360-11L could inhibit the transcription of IFN-β, ISG15 and ISG56 in PAMs after ASFV infection (Figure 7A), indicating that ASFV MGF360-11L could inhibit the IFN-I antiviral response, which was consistent with our previous study (Figure 2). The IFN-β, IL-1β and IL-6 levels in cell culture supernatants were measured by ELISA. The results indicated that ASFV MGF360-11L could inhibit the expression of IFN-β, IL-1β and IL-6 in ASFV-infected PAMs (Figure 7B). Further analysis revealed that compared with nontargeting control siRNA, siMGF360-11L inhibited P72 protein expression in ASFV-infected PAMs (Figure 7C).

## Discussion

ASF is a viral hemorrhagic infectious disease of pigs and wild boars that is caused by ASFV and has a fatality rate as high as 100% [25, 26]. There is currently a lack of effective vaccines to prevent and control this disease. Evasion of host innate immunity plays a vital role in the pathogenesis of ASFV. Innate immunity is the host’s first line of defense against pathogenic microorganisms [27, 28]. Inflammation and IFN-I are important components of the innate immune response. How ASFV escapes surveillance by the host innate immune system and the relationship between immune escape strategies and the pathogenicity of the virus are poorly understood. Therefore, we investigated whether the coding gene of the ASFV SY18 strain could mediate immune escape by inhibiting the expression of IFN-I mediated by cGAS-STING. The genes encoding the ASFV SY18 strain were screened by dual fluorescent reporter genes, and the results revealed that ASFV MGF360-11L has an inhibitory effect on IFN-β and ISRE promoter activity (Figures 1A–F).

To evade the host's innate immune response, ASFV encodes many proteins that inhibit the production of IFN-I by antagonizing cGAS-STING signaling. Previous studies have reported that ASFV MGF360 and MGF530/505 play important roles in inhibiting the IFN-I response and antiviral activity [29–31]. ASFV MGF360-11L is a member of ASFV MGF360 and may act as an interferon escape protein to inhibit the host innate immune response. Recent studies have suggested that the ASFV Armenia/07 strain can control the production of IFN-β through the cGAS-STING pathway [32]. Additional evidence has shown that China 2018/1 ASFV DP96R could inhibit the production of IFN-I via the cGAS-STING-TBK1 signaling pathway [18]. Moreover, ASFV MGF505-7R could also inhibit the production of IFN-I via the cGAS-STING signaling pathway [19]. In this study, ASFV MGF360-11L not only inhibited cGAS-STING-mediated activation of the IFN-β and ISRE promoters in a dose-dependent manner in HEK-293T cells but also significantly inhibited downstream IFN-I antiviral responses.

TBK1 is a key transcription factor in the cGAS-mediated signaling pathway, and its phosphorylation is essential for the activation of IRF3 and NF-κB and induction of IFN-β [33, 34]. IRF-7 is the most important regulator that induces the expression of IFN-I [35]. Studies have shown that the ASFV DP96R protein can suppress the antiviral immune response by reducing the phosphorylation of TBK1 [18]. ASFV MGF505-7R inhibited the phosphorylation of TBK1 and IRF3 induced by B-DNA transfection in PAMs [19]. In the present study, ASFV MGF360-11L interacted with TBK1 and IRF7 and degraded TBK1 and IRF7 through the cysteine, ubiquitin–proteasome and autophagy pathways. Moreover, ASFV MGF360-11L could also inhibit the IFN-I response by reducing the phosphorylation of TBK1 and IRF3. Mechanistically, we demonstrated that ASFV MGF360-11L could inhibit IFN-I production by binding to TBK1 and IRF7. Therefore, our results revealed another mechanism by which ASFV escaped host IFN-I signaling by directly targeting TBK1 and IRF7.

Innate immunity is the host's first line of defense against viral infection. When the body is invaded by pathogenic microorganisms, there are a series of pattern recognition receptors (PRRs) in the innate immune system that recognize virus-derived porcine alveolar macrophages (PAMs) and trigger a series of signals, causing the production of IFN and the inflammatory cytokines IL-1β and IL-6 to resist viral infection [36]. Recent studies have shown that pMGF505-7R can interact with NLRP3 to inhibit NLRP3 inflammasome assembly, leading to decreased IL-1β production [20]. Sendai virus V protein can inhibit the secretion of IL-1β by preventing NLRP3 inflammasome assembly [37]. In this study, ASFV MGF360-11L inhibited the production of the inflammatory factors IL-1β, IL-6 and IFN-β during ASFV infection. ASFV MGF360-11L can inhibit the translocation and activation of NF-κB, thereby limiting the synthesis of inflammatory factors (IL-1β and IL-6).

In conclusion, our results demonstrated that ASFV MGF360-11L inhibited IFNs and ISGs, blocked p-TBK1 and p-IRF3, interacted with TBK1 and IRF7, and degraded TBK1 and IRF7 by the cysteine and autophagy pathways to inhibit IFN-I-mediated antiviral activity. We will knock out the MGF360-11L gene and construct an ASFV strain with MGF 360-11L gene deletion to obtain safe and effective attenuated ASF vaccine candidate strains. This study provides potential strategies for the development of ASFV attenuated vaccines.

## Data Availability

All data generated or analyzed during this study are included in this published article.
